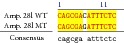# Genetic Diversity of *SCN5A* Gene and Its Possible Association with the Concealed Form of Brugada Syndrome Development in Polish Group of Patients

**DOI:** 10.1155/2014/462609

**Published:** 2014-10-20

**Authors:** Beata Uziębło-Życzkowska, Grzegorz Gielerak, Paweł Siedlecki, Beata Pająk

**Affiliations:** ^1^Department of Cardiology and Internal Diseases, Military Institute of Medicine, Szaserów Street 128, 04-141 Warsaw 44, Poland; ^2^Department of Bioinformatics, Institute of Biochemistry and Biophysics, Polish Academy of Sciences, 02-106 Warsaw, Poland; ^3^BioVectis/Kucharczyk TE, Pawinskiego 5A, 02-106 Warsaw, Poland; ^4^Electron Microscopy Platform, Mossakowski Medical Research Centre, Polish Academy of Sciences, 02-106 Warsaw, Poland

## Abstract

Brugada Syndrome (BS) is an inherited channelopathy associated with a high incidence of sudden cardiac death. The paper presents the discovery of new genetic variants of *SCN5A* gene which might be associated with the development of a concealed form of Brugada Syndrome. The study involved a group of 59 patients (37 men) with suspected concealed form of Brugada Syndrome. Pharmacological provocation with intravenous ajmaline administration was performed. Six patients with positive test results were subjected to molecular analysis of *SCN5A* gene with MSSCP method. Additionally, MSSCP genotyping was performed for samples obtained from the family members with Brugada Syndrome, despite the fact that they had negative ajmaline challenge test results. Genetic examinations of the *SCN5A* gene at 6 positive patients showed 6 known polymorphisms, 8 new single nucleotide point (SNP) variants located at exons, and 12 new single nucleotide point variants located at introns. Among new SNPs localized in *SCN5A* gene exons three SNPs affected the protein sequence.

## 1. Introduction

About 5–10% of sudden cardiac death (SCD) cases are caused mainly by electrical heart diseases [1]. In the recent years, a special attention has been paid to one of them, the Brugada Syndrome (BS). The diagnosis of BS is based on ECG criteria as well as on the clinical picture. Typical BS ECG changes occur as a result of ion function disorders in the heart, which are caused by genetic mutations and lead to improper course mainly of repolarization processes in cardiomyocytes. Electrocardiographic features of the syndrome are dynamic and ECG curve is periodically normal; typical BS characteristics disappear, which makes it difficult to diagnose BS. Concealed form of BS causes underestimation of this disease's occurrence frequency and a great number of people remain undiagnosed. Specific pharmacological provocation tests with class I medicine are critical in revealing concealed ECG features of BS.

### 1.1. Genetic Background of the BS

The first gene to be linked to BS is the* SCN5A*, the gene that encodes the *α*-subunit of the cardiac sodium channel gene [2]. Almost 400 mutations at the* SCN5A* gene have been identified at the syndrome patients since 2001 [3, 4]. Numerous detected mutations have been studied at the functional level [5]. The mutations at the* SCN5A* gene occur in approximately 18% to 30% of Brugada Syndrome cases. A higher incidence of* SCN5A* mutations has been reported in familial rather than in sporadic cases [6].

Other gene loci on chromosome 3, which is close to but distinct from* SCN5A*, have recently been linked to the syndrome (3p22–p24) [6] and GPD-1L [7]. Those mutations resulted in the loss of function of the cardiac sodium channel. Other genes associated with BS were reported in the last few years and shown to encode the *α*1 and *β* subunits of the L-type cardiac calcium channel [8].

The* SCN5A* gene remains the main gene linked to BS. Of note, negative* SCN5A* results generally do not rule out causal gene mutations. Currently, knowledge of a specific mutation may not provide guidance in formulating a diagnosis or determining a prognosis. Mutation screening of the* SCN5A* gene in patients with BS may only support a clinical overt or suspicious diagnosis.

In recent years, the genotyping of* SCN5A* gene was more correlated to the prognostic value than to the diagnosis of the BS itself. Some of the* SCN5A* mutations were related to a worse clinical course [9] and others to a better [10] prognosis of the BS patients.

## 2. Materials and Methods

### 2.1. Patient Populations

The study involved a group of 59 Polish patients (37 men) with suspected concealed BS based on specific ECG and/or clinical criteria:

- complete and incomplete right bundle branch block (RBBB) in ECG,
- suspected but nondiagnostic ECG (types 2 and 3),
- history of sudden cardiac arrest (SCA),
- unexplained syncopes,
- sudden cardiac death (SCD) amongst family members under 45,
- family history of BS.

The protocol of the study has been approved by the Commission for Bioethics. Written informed consent was obtained from all of the patients.

### 2.2. The Ajmaline Challenge Test

All patients were performed with pharmacological provocation with intravenous ajmaline administration dosed 1 mg/kg body weight for 5 min, in safe conditions during 12-lead 24-hour Holter ECG monitoring.

In patients with positive test results, molecular tests of the* SCN5A* gene were performed.

Molecular tests were performed in the family members of patients with BS, even if the pharmacological provocation test was negative in these individuals.

Occurrence of type 1 electrocardiographic patterns (cove-shaped ST elevation in right precordial leads with J wave or ST elevation of ≥2 mm (mV) at its peak followed by a negative T wave with little or no isoelectric interval in more than one right precordial lead, V1–V3) or conversion of type 2 or 3 to the diagnostic type 1 pattern after ajmaline administration was considered as a positive test result [3]. Occurrence of type 2 or 3 ST segment elevation was considered as negative test result.

### 2.3. DNA Analysis

The genetic analysis was conducted in collaboration with Kucharczyk TE/BioVectis Company (Warsaw, Poland). Genomic DNA were analysed in 7 patients with positive result of ajmaline challenge test (one patient with positive result of ajmaline challenge test did not agree to be genotyped) and in 1 family member of patients with negative result of ajmaline challenge test. Genomic DNA were extracted from peripheral blood leucocytes (100 *μ*L of frozen blood was used). Isolation was performed according to the manufacturer's protocol (A&A Biotechnology, Poland). Regions most likely to contain genetic mutations at 28 exons of the* SCN5A* gene were covered by 41 PCR amplicons, covering 28 exons and partial intron sequences, as previously described [11]. Several pairs of primers were synthesized to amplify with PCR reaction exons 12, 17, and 28 due to their large sizes, named as 12a, 12b, 17a, and so forth. PCR primers were designed to cover the full coding sequence (exons), as well as partial fragments of flanking noncoding fragments (introns). The PCR products were separated on agarose gel to examine their specificity and to normalise the DNA concentration. Next, 328 PCR products were screened by multitemperature single-strand conformation polymorphism (MSSCP) [12] method for the presence of a single-point mutation or a polymorphism. The MSSCP conditions were individually optimized for each PCR product. MSSCP was performed on 7 to 10% T polyacrylamide gel, 3.3% C at 0.75x TBE buffer. For some regions, glycerol was added to polyacrylamide gel up to 5% w/v concentration. MSSCP analysis was performed using DNA* Pointer* System in 0.5x TBE buffer. Temperature profile of electrophoresis was 35–15–5°C. Electrophoresis was performed with 40 W of electrical power. Before applying samples onto the gel, 10 min of preelectrophoresis (40 W at 35°C) was performed. At the beginning, samples were maintained for 10 min at 100 V for concentration. Subsequently, MSSCP separation was made. The PCR products that have altered MSSCP mobility were followed by Sanger method. 20 ng DNA of PCR products were used as a matrix for sequencing reaction. Both strands were sequenced at PCR products that revealed a genetic alternation. Genetic alterations were identified using the BLAST (Basic Local Alignment Search Tool) program and its BLASTN version as well as UCSC (University of California Santa Cruz) Genome Bioinformatics and NCBI (National Center for Biotechnology Information) databases of single-nucleotide polymorphisms (SNPs).

### 2.4. Functional Analysis of* SCN5A* Variants

An in silico analysis was performed to evaluate the putative functional impact of the three identified variations (S321Y, S519F, and K974D). We used the Polymorphism phenotyping-2 (PolyPhen-2) server [13], which integrates sequence-based and structure-based features to predict amino acid substitution effects using a naïve Bayes classifier. An amino acid change was classified as “probably damaging” if its probability score was greater than 0.85 or as “possibly damaging” if the score was between 0.85 and 0.55. To assess the influence of putative unstructured regions, we used DISOPRED3 [14] software along with DOMPRED [15] to predict possible domain boundaries and disordered binding regions. Finally, we used Phyre [16] for structural feature predictions, mainly transmembrane regions and secondary structure using three different algorithms.

## 3. Results

### 3.1. Patient Demographics

Study inclusion criteria were met by 59 patients (22 women and 37 men) (Table 1). Average age of the group was 31.6 ± 12.2 years, from 16 to 62 years. Average age for women was 29.68 ± 10.9 years while, for men, it was 32.8 ± 12.9 years. The majority of patients (72.8%) were under 40.

Echocardiography in all the included patients revealed no significant organic heart disease.

### 3.2. Clinical Characteristics of the Group with Positive Result of Pharmacological Provocation Test

Pharmacological provocation test was carried out on the whole study group. No significant undesirable effects were observed. None of the patients met the criteria of discontinuation prior the scheduled conclusion of the study.

**Figure 1:**
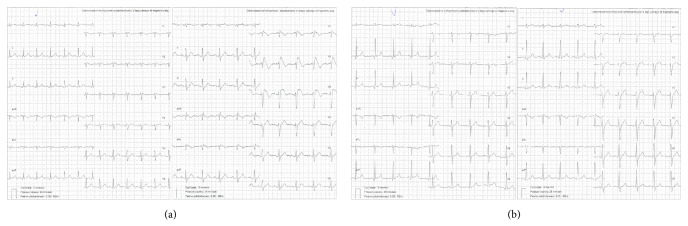
(a) 12-lead ECG from a patient with positive test result (before and after test). The configuration of the ST segment elevation in leads V1 to V3 is a coved type. (b) 12-lead ECG from a patient with negative test result (before and after test).

Positive test result—type 1 ST segment elevation (Figure 1(a)), which was considered as diagnostic of BS, was obtained in 7 individuals (11.86%). The other 52 patients (88.14%) had negative provocation test results (Figure 1(b)).

The group of 7 patients with type 1 ST segment elevation diagnostic of BS following ajmaline administration consisted of 6 men (85.7%) and 1 woman (14.3%). Average age of this group was 36.5 ± 15.2, from 16 to 52 years. The group of patients with negative test results included 31 men (59.6%) and 21 women (40.4%). Average age of this group was 30.9 ± 11.7, from 18 to 62 years. No statistically significant correlation between gender, age, or body mass and ajmaline test was observed.

As regards the group of 7 individuals with positive provocation test results, 2 patients had history of SCA (men), among whom, in 1 person, the diagnosed SCA mechanism was ventricular fibrillation. The SCA mechanism in the second individual remains unknown. Both patients were implanted a cardioverter-defibrillator. Within the group of the other 5 patients, initially considered as asymptomatic, 16 months following the provocation test, syncopes occurred in 1 person (woman), which was an indication of implanting a cardioverter-defibrillator. The other 4 individuals have remained asymptomatic during the observation period lasting from 39 to 60 months.

### 3.3. Results of MSSCP Analysis and DNA Sequencing of the* SCN5A* Sodium Gene

Genetic examinations of* SCN5A* gene showed 6 known polymorphisms: rs6599230 (A>G, A29A), rs41312393 (A>G, intron), rs1805126 (T>C, A1818G), rs7429945 (A>G, exon, nontraslanted region), rs41315485 (T>C, exon, nontraslanted region), and rs7430407 (A>G, E1061E). Three of them were noted at regions of coding proteins, two at noncoding regions and one at intron. Numerous new genetic variants were detected: at nontraslanted regions (8 SNPs), at introns (12 SNPs), and in the protein coding regions (5 SNPs)—2 DNA sequence variants caused no change in the coded amino acid, whereas 3 altered the coded amino acid.

**Figure 2:**
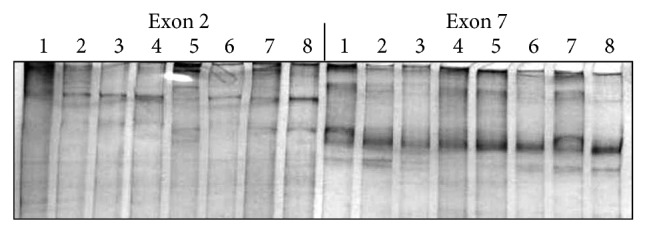
MSSCP separation of exon 2 and exon 7 PCR products. Note that samples number 5 and number 7 at exon 2 and sample number 1 at exon 7 have distinct electrophoretic profiles suggesting the presence of minor genetic variants.

**Figure 3:**
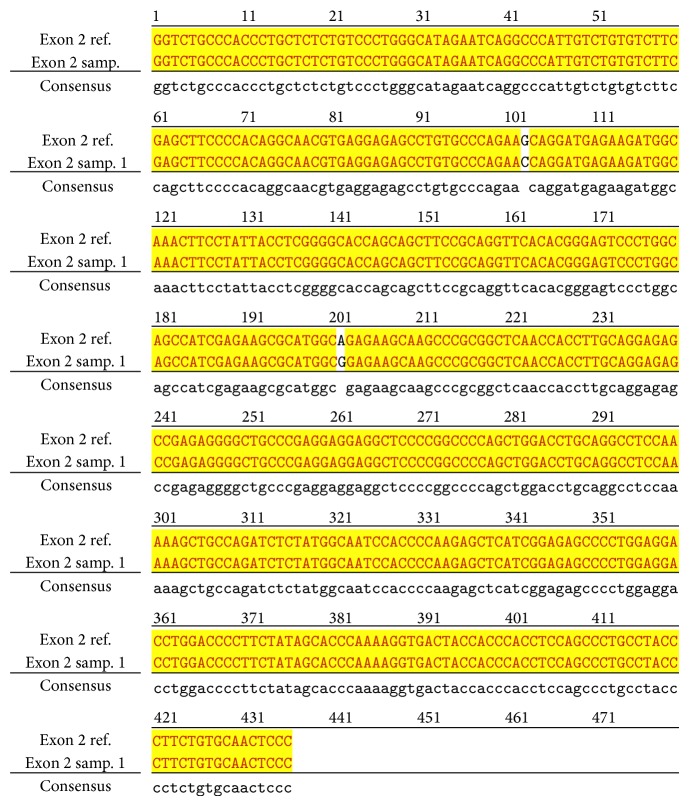
Sequence alignment of* SCN5* exon 2 reference (WT) and MT amplicon detected in sample 5 (pos. 38614716 heterozygote A>G, exon, amino acid pos. 29, polymorphism rs6599230; pos. 38614815 heterozygote G>C, exon, pos. 182 in mRNA, nontraslanted region). White color shows changed nucleotides.

An example of MSSCP analysis of 2 amplicones representing exons number 2 and 7 of the* SCN5A* gene for 8 particular patients is presented in Figure 2. On the other hand, Figure 2 shows an example of patient's derieved-amplicone sequence analysis, which was compared with reference sequences (Figure 3). All detected polymorphisms were further analyzed in context of their localization and its impact on aa SCN5A protein sequence.

### 3.4. Known Polymorphisms

The rs6599230 polymorphism at exon 2 of the* SCN5A* gene was found in 2 patients related to each other. It involved an alteration of nucleotides in 38614716 position (A>G) of reference sequence; however, detected variant did not alter the aa in protein sequence (A29A), thus having no impact on protein function. The patients with this variant were a man (father) and a woman (daughter), both asymptomatic. The pharmacological provocation test was positive in the man and negative in the woman.

On the other hand, in exon 17b of the* SCN5A* gene, a known rs7430407 polymorphism was identified in 1 person. It involved a nucleotide alteration in 38562471 position (A>G) of reference sequence. The patient with this variant was a man with asymptomatic BS diagnosed based on pharmacological provocation. This genetic alteration caused no amino acid changes in protein sequence (E1061E).

Genotyping exon 24 of the* SCN5A* gene revealed a known rs41312393 polymorphism in 3 individuals. It involved an alteration in nucleotides in 38538672 position (A>G) of reference sequence and was located at intron. The 3 persons were asymptomatic—2 men with positive pharmacological provocation test and a woman related to one of the men (daughter) with a negative result.

At exon 28c of the* SCN5A* gene a known 1805126 polymorphism was identified in 4 patients. This genetic change involved a nucleotide alteration in 38532410 position (T>C) of reference sequence and caused no change in the amino acid sequence in the coded protein (D1818D). Clinically, there were 2 asymptomatic individuals related to each other (father and son); one had negative result of pharmacological provocation (father) whereas the second patient had type 1 change in ST segment typical of BS. The other 2 patients were not related; one was a man with symptomatic BS and with history of SCA while the other was a man with asymptomatic BS.

Further, analysis of exon 28f of the* SCN5A* gene revealed the presence of known rs7429945 polymorphism, which was detected in 7 patients. It involved a nucleotide alteration in 38531693 position (A>G) of reference sequence. This genetic change occurred in the nontraslanted part of the exon in 6365 mRNA position. The described genetic change was present in almost every patient. Its presence was not observed only in a man with symptomatic BS and history of SCA.

Another known polymorphism is rs41315485 identified in 6 patients at exon 28k of the* SCN5A* gene. It involved an alteration in nucleotides in 38530279 position (T>C) of reference sequence, in 7779 mRNA position, and was located in the nontraslanted region. The polymorphism was not observed only in 2 individuals from the analyzed group. They were men (brothers)—one with symptomatic BS and the other with asymptomatic BS.

### 3.5. New Genetic Variants in Nontraslanted Regions at Exons

At exon 1 of the* SCN5A* gene, a new polymorphism that involved an alteration in nucleotides in 38631119 position (G>A) of reference sequence in 49 mRNA position was observed. The change was connected with the region transcribed on mRNA but is not translated as a protein. The person with this genetic variant was a man with diagnosed symptomatic BS (with history of SCA). The polymorphism was not observed in other patients.

Another new DNA sequence change was observed in 2 patients who were related to each other. It was connected with the change in nucleotides in 38614815 position (G>C) of reference sequence found at exon 2. The genetic variant was present in 182 mRNA position and was related to a nontraslanted mRNA part. The patients with this polymorphism (a woman and a man) were asymptomatic; pharmacological provocation test was positive in the man and negative in the woman.

A new DNA sequence change was also observed at exon 28g of the* SCN5A* gene in 5 patients. It involved an alteration in nucleotides in 38531355 position (G>A) of reference sequence in 6703 mRNA position. The genetic alteration was present in a nontraslanted part of the exon. The 5 individuals included 2 men with symptomatic BS and history of SCA. Moreover, the group included the brother and the father of the patient with history of SCA, one with a negative and the other with a positive result of pharmacological provocation test. The last person with this polymorphism was a man with asymptomatic BS.

Genotyping exon 28i of the* SCN5A* gene revealed 4 new polymorphisms localized in a nontraslanted part of the exon. These changes were found in all the examined persons, among whom 4 patients had all the 4 genetic variants and 3 patients had two new sequence changes: in 38530974 (C>T) and 38531102 (C>T) position of reference sequence, while polymorphism in 38530974 position (C>T) was observed in all the patients.

Furthermore, analysis of exon 28l of the* SCN5A* gene showed a new sequence variant in 38529996 position (C>G) of reference sequence in 8062 mRNA position. The change was found only in 1 person. Clinically, the person with this polymorphism was a man with asymptomatic BS.

### 3.6. New Polymorphisms in Protein Coding Regions That Cause No Alteration in the Coded Amino Acid

As regards the group of 8 examined patients, 2 unknown genetic variants were observed in one patient at exon 28c of the* SCN5A* gene in protein coding regions; they caused no change in the coded amino acid; thus, we should consider them a polymorphic change. The two novel polymorphic variants were detected in positions 38532614 (C>T, F1750F) and 38532617 (C>T, L1749L). In both cases, the changed nucleotide is in the 3rd coded position, which may influence the fact that it causes no alteration in the amino acid sequence. These genetic variants were observed in a patient with diagnosed BS and history of SCA who required implantation of cardioverter-defibrillator.

### 3.7. New Sequence Changes in the Protein Coding Regions That Alter the Coded Amino Acid

During genetic analysis of the* SCN5A* gene, presence of 3 unknown genetic variants that altered the coded amino acid was noticed in 5 patients.

The first variant contained change in nucleotides in 38589682 position (C>A) of reference sequence and was observed in 1 patient with negative result of pharmacological provocation. This genetic change was observed in exon 8 of the* SCN5A* gene. It altered serine amino acids into tyrosine in 321 position of the coded protein (S321Y). This variant was found in none of the other patients.

Another genetic variant detected in one patient involved a change in nucleotides in 38585541 position (C>T) of reference sequence, which altered serine amino acids into phenylalanine in 519 protein position (S519F). New variant was found at exon 12 of the analyzed gene. The change was observed in a patient with asymptomatic BS and its presence was confirmed neither in 2 family members of the patient nor in the other examined patients.

**Figure 4:**
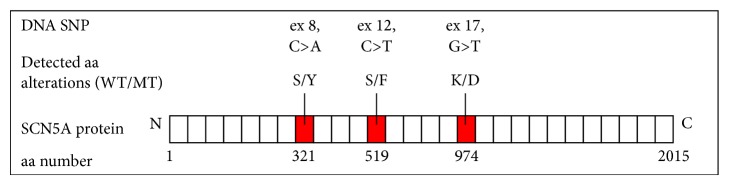
Schematic illustration of Nav1.5, showing the location of the novel putative amino acid changes.

The last new sequence variant, which, according to the UCSC Genomi Bioinformatic database, is found in the protein coding region, was observed in 4 patients. The polymorphism was observed at exon 17 of the* SCN5A* gene and involved a change in amino acids in 38562732 position (G>T) of reference sequence. This genetic change altered lysine amino acids into aspartic acid in 974 position of the coded protein (K974D). BS was diagnosed in 2 of the persons while the other 2 individuals were family members of patients with negative results of pharmacological provocation. Schematic representation of detected changes is illustrated in Figure 4. To evaluate the possible influence of new missense mutations on channel function, bioinformatics analysis has been conducted.

### 3.8. In Silico Functional Analysis of* SCN5A* Variants

**Figure 5:**
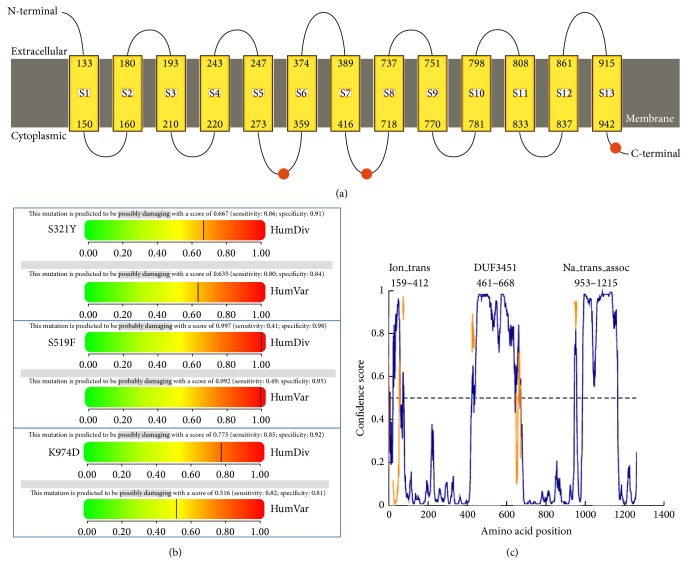
(a) Prediction of transmembrane, extracellular in cytoplasmic regions in SCN5a protein. Location of the three new variants is depicted as orange dots. (b) Prediction of functional effects of nonsynonymous mutations done in PolyPhen2 software. All three variants have two scores from HumDiv (genomic oriented) and HumVar (diagnostic oriented). (c) Prediction of intrinsically disordered regions in SCN5a (1–1400AA) done by DISOPRED3. Domain organisation shown with respect to disordered regions. Orange color marks putative binding sites. High confidence score indicated better chance of unstructured fragment.

The three variants identified are located at the cytoplasmic region of the* SCN5A*-encoded protein (Figure 5(a)). Confirmed disease associated genetic variants can be found in close proximity to each one of the new variants, as well as sites of amino acid modifications (e.g., arginine methylation site at 513 and 526 or a glycosylation site at 318). This would hint that the observed variants are located in important regions for protein function. To further explore their possible functional impact, we employed a well-known bioinformatics algorithm PolyPhen2. The tool indicated a high possibility of damage caused by mutating K974D with prediction score close to 1 (the highest possible) (Figure 5(b)). For the two other mutations, possible damage was also reported, but with lesser probability. We sought to confirm these predictions with more structural insights. We used three different software tools to establish whether these variations would occur in unstructured and putative domain regions or not. Indeed, S519F is located in a large domain of unknown function (DUF3451, PFAM: PF11933), which is also predicted as an unstructured/disorderd region by all three bioinformatics methods (Figure 5(c)). This would suggest a possible protein binding interaction in this region, which could be hampered by this variant (especially since serine contains a hydroxylic polar group and phenylalanine is hydrophobic and aromatic). On the other hand, S321Y is also located in transmembrane ion channel family domain (Ion_trans, PF) which is predicted to be structured. The same goes with K974D, located just at the beginning of the sodium ion transport-associated domain (Na_trans_assoc, PF06512). Again, this is a structured region but very close to the predicted unstructured binding region (945–956).

### 3.9. New Point Mutations Found at Introns

In the regions of the* SCN5A* gene, which, according to the UCSC Genome Bioinformatics database, are at introns, 12 new point mutations were found.

MSSCP analysis of exon 4 of the* SCN5A* gene detected 4 new mutations within the intron. The first was a mutation in 38603806 position of reference sequence and involved a type A insertion. The second mutation was an alteration in nucleotides in 38603801 position (T>A) of reference sequence. The two said genetic changes were confirmed in 5 patients, among whom 3 were asymptomatic and 2 were symptomatic (1 with history of SCA and 1 with syncopes). Another genetic change detected at this exon in the other 2 individuals was a change in nucleotides in 38604076 position (G>T) of reference sequence. The first patient had positive provocation result and history of SCA whereas the second patient was asymptomatic and also had positive provocation result. The last sequence change at this exon was found only in one patient in 38604075 position (G>T) of reference sequence. The patient was asymptomatic with negative result of ajmaline test.

MSSCP analysis showed presence of 2 new genetic variants at exon 6 of the* SCN5A* gene within the intron in 7 patients out of 8. The first involved a change in nucleotides in 38595390 position (C>G) of reference sequence. The second mutation was a change in 38595384 position (C>G) of reference sequence. These DNA variants were observed in all the examined patients apart from one individual who was asymptomatic and had negative result of pharmacological provocation test. At exon 7 of the* SCN5A* gene, a DNA sequence variant within the intron (pos. 38591480, C>G) was identified in 4 patients out of 6 with positive ajmaline provocation test. Moreover, 3 patients from this group had symptomatic BS and had an implanted cardioverter-defibrillator either due to history of SCA or due to unexplained syncopes. Concurrently, this polymorphism was confirmed in neither of the patients with negative results of provocation test. The results of MSSCP genotyping of 41 amplicons representing* SCN5A* gene are summarized in Table 2. Additionally, Table 3 contains the list of intronic alterations and exchanges in noncoding regions along with short stretches of sequence alignments (WT > MT).

## 4. Discussion

The major gene related to BS is the* SCN5A* gene. Despite the great development in molecular studies, it is estimated that mutations in the* SCN5A* gene cause only about 18–30% of BS cases [17]. These mutations are more common in familial cases of the disease than in sporadic ones [18]. Negative results of genetic studies do not exclude causal gene mutations. Neither diagnosis nor prognosis of BS can be based on genetic test results. In the presented work, a molecular analysis of the whole* SCN5A* gene was carried out with respect to patients with positive provocation test (apart from 1 person who failed to give their informed consent) as well as their family members (1st degree of kinship) who gave their informed consent. Due to both low predicted BS incidence in the Polish population (lack of accurate data) and a considerably low percentage of the known genetic changes being the underlying cause of the disease (18–30% as above), the work was limited only to analyzing the occurrence of the known mutations. The molecular study of the 28 exons and short exon/intron fragments of* SCN5 *gene was carried out including also the alterations in the sequence of the few noncoding regions of the gene (introns). In this study, the new genetic variants were found both at exons and at introns. It is a commonly accepted fact that the effects of DNA sequence change depend on their location in the gene. However, all too often, it is assumed that only genetic alterations in the coding sequences, that is, at exons, have an impact on the clinical course of the disease. Recent studies and findings have shown that intronic mutations may play a major role in the splicing process, alter its course, lead to coding sequence abnormalities, and consequently influence the structure and function of the encoded proteins. Numerous data reported in scientific papers show that both intronic and exonic alterations may result in an aberrant splicing process, leading to the formation of abnormal proteins, which, in turn, affects the severity of the disease symptoms. These mutations/polymorphisms at introns leading to the disturbances of the splicing process are described in the disorders of cardiovascular system [19].

In the course of DNA analysis of the* SCN5A* sodium gene, the following 6 known polymorphisms were identified: rs6599230, rs41312393, rs1805126, rs7429945, rs41315485, and rs7430407. In this group, 3 polymorphisms were observed in the protein coding regions, 2 in the nontraslanted regions, and one at the intron. None of them had been associated with BS before. Also, 8 new genetic variants were found at exons in the nontraslanted regions, 12 at introns, and 2 in the protein coding regions that cause no change in the coded amino acid. None of 3 point mutations (S321Y, S519F, and K974D) in the protein coding regions that alter the coded amino acid has been associated previously with BS [5]. According to Zimmer and Surber, as well as bioinformatic analysis, we are able to localize their positions in protein sequence. S321Y is localized in the intracellular loop III, S519F in the intracellular loop IV, whereas K974D is localized in the C-terminal intracellular fragment of SCN5a protein. According to the bioinformatic results, K974D aa alteration is recognized as highly damaging for protein function (prediction score amounted to about 1, the highest possible). Two other aa changes were also reported as possible damage; however, their probability score amounted from 0.85 to 0.55.

As mentioned previously, also intronic changes could affect protein function. We performed some basic bioinformatic analyses of detected changes; however, we obtained contradictory data. Due to the large number of detected polymorphisms in introns, we decided to perform more detailed analyses, including* in vitro* studies.

The majority of detected polymorphisms and genetic changes found in the study had never been reported as mutations leading to development of BS. The lack of data in the literature and the lack of a population control for this part of the* SCN5A* gene made it impossible to state clearly whether the BS syndrome was significantly associated with the mentioned changes or not. It is also noteworthy that several genes are associated with BS syndrome; thus, further genetic study is needed. However, at least new polymorphisms/mutations that were found in our patients of a specific phenotype are worth considering.

Special attention ought to be paid to genetic changes present only in symptomatic patients, for example, with history of SCA. These genetic changes include the following:

- a new polymorphism which involves an alteration in nucleotides in 38631119 position (G>A) of reference sequence in 49 mRNA position and developed in a man with history of SCA and BS diagnosed on the basis of provocation test result; it was found in none of the other patients;
- a new genetic variant at exon 28 which involves an alteration in nucleotides in 38531355 position (G>A) of reference sequence in 6703 mRNA position and developed in 5 patients including 2 men with positive provocation test results and history of SCA as in other individuals (i.e., the brother and the father of one of these men);
- two new genetic variants at exon 28c in the protein coding regions with no alteration in the coded amino acid (C>T, I1749I; C>T, F1750F) both developed in a man with history of SCA and positive provocation test results;
- a new polymorphism at exon 7 which involves an alteration in nucleotides in 38591480 position (C>G) of reference sequence and developed in 4 patients out of 6 individuals with positive ajmaline provocation test, 3 of these patients had symptomatic BS following implantation of cardioverter-defibrillator either due to history of SCA or due to unexplained syncopes; concurrently the mutation was confirmed in none of the patients with negative provocation test result.

Considering new data on the role of genetic changes not only in BS diagnostics but also in prognosis for diagnosed patients [10, 20], further studies aimed at determining the role of the identified genetic disorders seem to be extremely interesting.

## 5. Conclusions

New genetic variants/polymorphisms in the* SCN5A* gene are present in patients with concealed forms of Brugada Syndrome, yet their role in pathogenesis requires further studies.

## Figures and Tables

**Table 1:** Distribution of the examined population depending on inclusion criteria.

Inclusion criteria	Number of included patients
RBBB in ECG (complete and incomplete)	35 patients (59.32%) (i) RBBB complete—8 patients (13.6%) (ii) RBBB incomplete—27 patients (45.76%)

History of SCA	7 patients (11.8%)

Unexplained syncopes	31 patients (52.5%)

SCD amongst family members under 45	5 patients (8.5%)

Family history of Brugada Syndrome	4 patients (6.8%)

Suspected but nondiagnostic ECG (types 2 and 3)	16 patients (27.11%) (i) Type 2—4 patients (6.78%) (ii) Type 3—12 patients (20.33%)

**Table 2:** The results of MSSCP genotyping of 41 amplicons representing *SCN5A* gene.

	Major clinical data	Genetic alterations in DNA sequences of *SCN5* amplicons (reference sequence ref|NT_022517.17|Hs3_22673)
Patient 1	Asymptomatic Negative pharmacological provocation test Father of the patient with diagnosed BS	Amplicon 4: pos. 38603806, insertion A, intron pos. 38603801 homozygote A, intron Amplicon 8: pos.38589643 insertion A, intron pos. 38589682 C>A, amino acid pos. 321, S>Y Amplicon. 17a: pos. 38562732 G>T, amino acid pos. 974, K>D Amplicon 28c: pos. 385924060 T>C, exon, amino acid pos. 1818 D>D, rs1805126 Amplicon 28f: pos. 38531693 A>G, exon, mRNA position 6365, near 3′UTR, nontraslanted region, rs7429945 Amplicon 28g: pos. 38531355 heterozygote G>A, mRNA position 6703, near 3′UTR, nontraslanted region Amplicon 28i: pos. 38530974 C>T, exon, mRNA position 7084, near 3′UTR, nontraslanted region pos. 38531102 C>T, exon, mRNA position 6956, near 3′UTR, nontraslanted region Amplicon 28k: pos. 38530279 T>C, exon, mRNA pos. 7779, 3′UTR, nontraslanted region, polymorphism rs41315485

Patient 2	History of sudden cardiac arrest Positive pharmacological provocation test Diagnosed BS	Amplicon 4: pos. 38603806, insertion A, intron pos. 38603801 homozygote T>A, intron Amplicon 6: pos. 38595390 G>C, intron pos. 38595384 homozygote G>C, intron Amplicone 7: pos. 38591480 C>G, intron Amplicon 28g: pos. 38531355 G>A, exon, mRNA position 6703, near 3′UTR, nontraslanted region Amplicon 28i: pos. 38530974 G>A, exon, mRNA position 7084, near 3′UTR, nontraslanted region

Patient 3	History of sudden cardiac arrest Positive pharmacological provocation test Diagnosed BS	Amplicon 1: pos. 38631119 G>A, mRNA pos. 49, nontraslanted region Amplicon 4: pos. 38604076 G>T, intron Amplicon 6: pos. 38595390 homozygote C>G, intron pos. 38595384 homozygote C>G, intron Amplicon 7: pos. 38591480 C>G, intron Amplicon 17a: pos. 38562732 G>T, amino acid pos. 974, K>D Amplicon 28c: pos. 38532617 C>T, exon, amino acid pos. 1749, I>I pos. 38532614 C>T, exon, amino acid position 1750, F>F pos. 38592406 T>C, exon, amino acid pos. 1818, D>D, rs1805126 Amplicon 28f: pos. 38531693 A>G, exon, mRNA position 6365, near 3′UTR, nontraslanted region, rs7429945 Amplicon 28g: pos. 38531355 heterozygote G/A, mRNA position 6703, near 3′UTR, nontraslanted region Amplicon 28i: pos. 38530974 C>T, exon, mRNA position 7084, near 3′UTR, nontraslanted region pos. 38531102 C>T, exon, mRNA position 6956, near 3′UTR, nontraslanted region Amplicon 28k: pos. 38530279 T>C, exon, mRNA pos. 7779, 3′UTR, nontraslanted region, polymorphism rs41315485

Patient 4	Asymptomatic Positive pharmacological provocation test Brother of the patient with diagnosed BS	Amplicon 4: pos. 38604076 G>T, intron Amplicon 6: pos. 38595390 homozygote C>G, intron pos. 38595384 homozygote C>G, intron Amplicon 12a: pos. 38585541 C>T exon, amino acid pos. 519, S>F pos. 38585647 homozygote A>T, intron Amplicon 22: pos. 38544050, heterozygote C/T, intron Amplicon 24: pos. 38547178 heterozygote A/G, intron, polymorphism rs41312393 Amplicon 28c: pos. 38592406 T>C, exon, amino acid pos. 1818, D>D, rs1805126 Amplicon 28f: pos. 38531693 A>G, exon, mRNA position 6365, near 3′UTR, nontraslanted region, rs7429945 Amplicon 28g: pos. 38531355 G>A, exon, mRNA position 6703, near 3′UTR, nontraslanted region Amplicon 28i: pos. 38530974 C>T, exon, mRNA position 7084, near 3′UTR, nontraslanted region pos. 38531102 C>T, exon, mRNA position 6956, near 3′UTR, nontraslanted region

Patient 5	Asymptomatic Positive pharmacological provocation test Father of the patient with diagnosed BS	Amplicone 2: pos. 38614716 A>G, exon, amino acid pos. 29, A>A, polymorphism rs6599230 pos. 38614815, G>C, exon, mRNA pos.182, nontraslanted region Amplicone 4: pos. 38604075 homozygote G>T, intron Amplicon 6: pos. 38595390 homozygote C>G, intron pos. 38595384 homozygote C>G, intron Amplicon 17a: pos. 38562732 G>T, amino acid pos. 974, K>D Amplicon 17b: pos. 38562471 homozygote A>G, amino acid pos. 1061, E>E, polymorphism rs7430407 Amplicon 24: pos. 38547178 heterozygote A>G, intron, polymorphism rs41312393 Amplicon 27: pos. 38536074 deletion T, intron pos. 38536077 homozygote T>A, intron Amplicon 28c: pos. 38592406 T>C, exon, amino acid pos. 1818, D>D, rs1805126 Amplicon 28f: pos. 38531693 A>G, exon, mRNA position 6365, near 3′UTR, nontraslanted region, rs7429945 Amplicon 28g: pos. 38531355 G>A, exon, mRNA position 6703, near 3′UTR, nontraslanted region Amplicon 28i: pos. 38530853 deletion C, exon, mRNA pos. 7205, near 3′UTR, nontraslanted region pos. 38530856 insertion A, exon, mRNA pos. 7202, near 3′UTR, nontraslanted region pos. 38530974 C>T, exon, mRNA pos. 7084, near 3′UTR, nontraslanted region pos. 38531102 C>T, exon, mRNA pos. 6956, near 3′UTR, nontraslanted region Amplicon 28k: pos. 38530279 T>C, exon, mRNA pos. 7779, 3′UTR, nontraslanted region, polymorphism rs41315485

Patient 6	Unexplained syncopes Positive pharmacological provocation test Diagnosed BS	Amplicon 4: pos. 38603806, insertion A, intron pos. 38603801 homozygote T>A, intron Amplicon 6: pos. 38595390 C>G, intron pos. 38595384 homozygote C>G, intron Amplicon 7: pos. 38591480 C>G, intron Amplicon 28f: pos. 38531693 A>G, exon, mRNA position 6365, near 3′UTR, nontraslanted region, rs7429945 Amplicon 28i: pos. 38530853 deletion C, exon, mRNA pos. 7205, near 3′UTR, nontraslanted region pos. 38530856 insertion A, exon, mRNA pos. 7202, near 3′UTR, nontraslanted region pos. 38530974 C>T, exon, mRNA pos. 7084, near 3′UTR, nontraslanted region pos. 38531102 C>T, exon, mRNA pos. 6956, near 3′UTR, nontraslanted region Amplicon 28k: pos. 38530279 T>C, exon, mRNA pos. 7779, 3′UTR, nontraslanted region, polymorphism rs41315485

Patient 7	Unexplained syncopes Positive pharmacological provocation test Diagnosed BS	Amplicon 2: pos. 38614716, A>G, amino acid pos. 29, A>A, polymorphism rs6599230 pos. 38614815, G>C, exon, mRNA pos.182, nontraslanted region Amplicon 4: pos. 38603806, insertion A, intron pos. 38603801 homozygote T>A, intron Amplicon 6: pos. 38595390 C>G, intron pos. 38595384 homozygote C>G, intron Amplicon 17a: pos. 38562732 G>T, amino acid pos. 974, K>D Amplicon 24: pos. 38547178 heterozygote A/G, intron, polymorphism rs41312393 Amplicon 28f: pos. 38531693 A>G, exon, mRNA position 6365, near 3′UTR, nontraslanted region, rs7429945 Amplicon 28i: pos. 38530853 deletion C, exon, mRNA pos. 7205, near 3′UTR, nontraslanted region pos. 38530856 insertion A, exon, mRNA pos. 7202, near 3′UTR, nontraslanted region pos. 38530974 C>T, exon, mRNA pos. 7084, near 3′UTR, nontraslanted region pos. 38531102 C>T, exon, mRNA pos. 6956, near 3′UTR, nontraslanted region Amplicon 28k: pos. 38530279 T>C, exon, mRNA pos. 7779, 3′UTR, nontraslanted region, polymorphism rs41315485

Patient 8	Asymptomatic Positive pharmacological provocation test	Amplicon 4: pos. 38603806, insertion A, intron pos. 38603801 homozygote T>A, intron Amplicon 6: pos. 38595390 C>G, intron pos. 38595384 homozygote C>G, intron Amplicon 7: pos. 38591480 C>G, intron Amplicon 28f: pos. 38531693 A>G, exon, mRNA position 6365, near 3′UTR, nontraslanted region, rs7429945 Amplicon 28i: pos. 38530853 deletion C, exon, mRNA pos. 7205, near 3′UTR, nontraslanted region pos. 38530856 insertion A, exon, mRNA pos. 7202, near 3′UTR, nontraslanted region pos. 38530974 C>T, exon, mRNA pos. 7084, near 3′UTR, nontraslanted region pos. 38531102 C>T, exon, mRNA pos. 6956, near 3′UTR, nontraslanted region Amplicon 28k: pos. 38530279 T>C, exon, mRNA pos. 7779, 3′UTR, nontraslanted region, polymorphism rs41315485 Amplicon 28l: pos. 38529996 homozygote C>G, exon, mRNA pos. 8062, near 3′UTR, nontraslanted region

**Table 3:** The list of intronic alterations and exchanges in noncoding regions along with short stretches of sequences alignments (WT > MT).

Amplicon	Position	Detected polymorphism (WT > MT)	Localization	Partial alignment (WT > MT)
1	Pos. 38631119	G>A	Exon, nontraslanted region	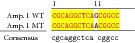

2	Pos. 38614815	G>C	Exon, nontraslanted region	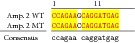

4	Pos. 38603806 Pos. 38603801	Insertion A T>A	Intron	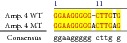

4	Pos. 38604075	G>T	Intron	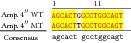

4	Pos. 38604076	G>T	Intron	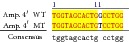

6	Pos. 38595390 Pos. 38595384	G>C G/C	Intron	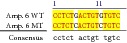

7	Pos. 38591480	C>G	Intron	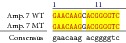

8	Pos. 38589643	Insertion A	Intron	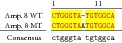

12	Pos. 38585647	A>T	Intron	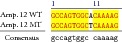

22	Pos. 38544050	C>T	Intron	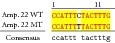

24	Pos. 38538672	A>G	Intron	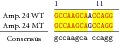

27	Pos. 38536074 Pos. 38536077	Deletion T T>A	Intron	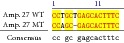

28f	Pos. 38531693	A>G	Exon, near 3′UTR, nontraslanted region	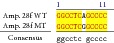

28g	Pos. 38531355	G>A	Exon, near 3′UTR, nontraslanted region	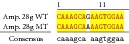

28i	Pos. 38530853 Pos. 38530856 Pos. 38530974 Pos. 38531102	Deletion C insertion A C>T C>T	Exon, near 3′UTR, nontraslanted region	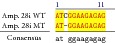 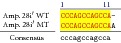 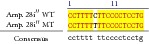 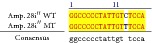

28k	Pos. 38530279	T>C	3′UTR, nontraslanted region	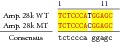

28l	Pos. 38529996	C>G	Exon, near 3′UTR, nontraslanted region